# Moral Bio-enhancement, Freedom, Value and the Parity Principle

**DOI:** 10.1007/s11245-017-9482-8

**Published:** 2017-04-12

**Authors:** Jonathan Pugh

**Affiliations:** Oxford Uehiro Centre for Practical Ethics, Suite 8, Littlegate House, St Ebbes Street, Oxford, OX1 1PT UK

**Keywords:** Freedom, Moral enhancement, Autonomy, Value

## Abstract

A prominent objection to non-cognitive moral bio-enhancements (NCMBEs) is that they would compromise the recipient’s ‘freedom to fall’. I begin by discussing some ambiguities in this objection, before outlining an Aristotelian reading of it. I suggest that this reading may help to forestall Persson and Savulescu’s ‘God-Machine’ criticism; however, I suggest that the objection still faces the problem of explaining why the value of moral conformity is insufficient to outweigh the value of the freedom to fall itself. I also question whether the objection is compatible with Neil Levy’s parity principle. Accordingly, I go on to consider an alternative relational freedom-based objection to NCMBEs that aims to explain the fundamental moral importance of the freedom that NCMBEs would violate. I argue that although this strategy might allow the critic of NCMBEs to bypass a powerful criticism of Harris’ objection, it also weakens the freedom-based objection’s compatibility with the parity principle.

Advocates of moral enhancement have argued that emerging biotechnologies could be used to modify moral behaviour. Such interventions might include cognitive bio-enhancements that serve to improve the recipient’s ability to understand and process morally relevant information. More controversially, some bio-enhancements might serve to modify moral behaviour by directly modulating emotions and motivational states (Douglas 2008, 2013a; Savulescu and Persson 2012). A prominent objection to the use of these latter non-cognitive moral bio-enhancements (henceforth NCMBEs), is that they would unacceptably compromise the recipient’s ‘freedom to fall’ (Harris 2011). Part of my aim in this paper is to resolve some ambiguities surrounding the nature of the freedom to fall. At this point, we may broadly understand this freedom to amount to the freedom to choose to act immorally, and to actually perform, immoral actions. In turn, this freedom is understood to be highly valuable (Harris 2011, 2013, 2016).

The ‘freedom to fall’ objection has been criticised by both advocates and critics of the general moral enhancement project (Douglas 2013a; Bublitz 2016; DeGrazia 2014; Hauskeller 2017; Persson and Savulescu 2015). My first aim in this paper is to show how discussions of this objection often invoke different understandings of what the ‘freedom to fall’ amounts to. I shall suggest that this is partly due to the fact that Harris at times takes the freedom to fall to refer primarily to the freedom to *do* immoral things, and at other times, the freedom to *choose* to do immoral things. I shall claim that one can resolve the ambiguities in Harris’ text by adopting an Aristotelian reading of the ‘freedom to fall’ objection, and that this reading might help to forestall at least one important criticism of the objection.

However, Harris’ objection still faces two important problems. First, despite Harris’ arguments to the contrary, there is room for scepticism about whether his objection is compatible with Neil Levy’s parity principle. The second problem is that the objection fails to adequately explain why the value of the freedom to fall should be understood to outweigh the value of the moral conformity that NCMBEs might achieve, where an agent is understood to conform to morality if her conduct coincides with her moral reasons to do (or refrain from doing) certain things (Douglas 2013b, 75). The Aristotelian reading I present may plausibly offer a response to the second problem; however I shall suggest that it relies on some problematic claims. As such, in the second part of the paper, I consider an alternative freedom-based objection to NCMBEs that appeals to *relational* freedom. The objection from relational freedom that I consider takes seriously the thought that a freedom-based objection to NCMBEs must render plausible the idea that allowing immoral actions is a price worth paying for preserving freedom. I shall argue that whilst this strategy might allow the objection from relational freedom to bypass an important criticism of the freedom to fall objection, it also raises significant doubts about its compatibility with the parity principle. I conclude by suggesting that considerations pertaining to the parity principle and the moral value of freedom lead freedom-based objections to NCMBEs into a dilemma.

I shall begin by elucidating Harris’ freedom to fall objection. Prior to doing so though, it is necessary to make the following terminological stipulation. Throughout the remainder of the paper, when I refer to the use of NCMBEs, I mean to refer solely to their *non-consensual* use unless stated otherwise; I take this to include both the enforced mandatory use of NCMBEs, and the use of NCMBEs in compliance with a coercive proposal. For the sake of brevity I shall omit this qualification in the remainder of the paper. I focus on non-consensual NCMBEs on the assumption that freedom-based objections to NCMBEs are likely to have greater force with respect to non-consensual NCMBEs than they do with respect to consensual NCMBEs.

## Two Ambiguities Concerning the Freedom to Fall

Harris’ discussion of the freedom to fall objection is somewhat lyrical, and it can be challenging to render his concerns into precise terms (Bublitz 2016). I suggest that this is partly a result of two ambiguities that are evident in Harris’ writings on the subject. The first ambiguity pertains to whether we should understand him to be claiming that the freedom to fall refers to a kind of freedom of action, which we may broadly understand as the freedom to perform a particular act, or to a kind of freedom of choice, which we may broadly understand as the freedom to decide what sort of actions to perform.

To illustrate this ambiguity, consider first that Harris at times seems to be concerned primarily with the threat that NCMBEs pose to the recipient’s freedom of action. He writes:

There are substantial issues of liberty which would need to be resolved and which could conceivably be threatened by any measures that make the freedom to *do* immoral things impossible (Harris 2011, 105, emphasis added).

Similarly, in other work, Harris criticises Tom Douglas for countenancing “making the unacceptable *undoable*” (Harris 2013, 170, emphasis added).

Yet, elsewhere in his delineation of the freedom to fall objection, Harris seems to be concerned primarily with the threat that NCMBEs pose to the recipient’s freedom of choice. For instance, in discussing Milton’s God’s pronouncement in *Paradise Lost*, that she has made man ‘sufficient to have stood, though free to fall’, Harris notes that Milton’s God is:

… underlining the sort of existential freedom … which allows us the exhilaration and joy of *choosing* (Harris 2011, 103, emphasis added).

Finally, in another passage, Harris invokes the language of autonomy, claiming that autonomy requires “… not only the possibility of falling but the freedom to choose to fall” (Harris 2011, 103). Here the suggestion seems to be that the freedom to fall should be understood to incorporate *both* freedom of action to do immoral things (i.e. freedom that allows for the possibility of falling), and freedom of choice to choose to perform those actions.

The ambiguous nature of Harris’ treatment of freedom is perhaps unsurprising, given the various understandings of this concept in the philosophical literature. This ambiguity is arguably exacerbated by the close relationship that Harris understands the freedom to fall to have with autonomy, as the quotation above suggests. The language of autonomy is commonly invoked in bioethical contexts, and is often understood to incorporate both freedom of choice and action in those contexts (Brock 1993, 29). On such an understanding, Harris’ discussion of ‘freedom to fall’ may be understood as a discussion of personal autonomy in the particular context of moral agency. However, freedom of action and freedom of choice have also been discussed separately in other contexts. For instance, what I have termed freedom of action may be at least partly be cashed out in terms of negative liberty, a central focus in political theory, particularly in discussions of libertarianism (Nozick 1974; Narveson 1988). In contrast, ‘freedom of choice’ is often understood to be closely related to the metaphysical debate surrounding free will, causal determinism and moral responsibility (Levy and McKenna 2009). To further complicate matters, some theorists in this latter area sometimes use the language of autonomy and moral responsibility in a seemingly interchangeable fashion, a tendency that Mariana Oshana (2002) has criticized.^1^ I lack the space to disentangle these various understandings here. For the purpose of my discussion, I shall begin with what I take to be Harris understanding of the relationship between autonomy and freedom; on this understanding, autonomy requires both freedom of choice *and* freedom of action. I shall say more about his understanding of the relationship between these freedoms and moral responsibility below.

Despite the fact that autonomy incorporates both of these freedoms on Harris’ understanding, there are some salient differences between freedom of action and freedom of choice. This is most evident when we consider the fact that certain interventions might affect one sort of freedom without directly affecting the other. Consider first an intervention that would limit only the recipient’s freedom of action. Such an intervention might have a similar effect to the fictional Ludovico technique that Anthony Burgess describes in his novel *A Clockwork Orange*. In the novel, the protagonist Alex is a highly violent individual who undergoes an extreme form of aversion therapy; the result of the therapy is that when Alex begins to form an intention to perform a violent act following the intervention, he becomes nauseous and finds himself unable to carry it out.

Contrary to this analysis, the example of the Ludovico technique has frequently been diagnosed as an intervention that undermines Alex’s freedom of choice. This is understandable, because it is the way that Burgess himself, through the character of the chaplain in the novel, describes the intervention (Burgess 2000). However, as McMillan (forthcoming) has recently explained, the chaplain seems to simply make a ‘factual error’ here.^2^ To illustrate the point, we may compare the Ludovico technique to an intervention that *would* infringe the agent’s freedom of choice (call this the ‘revised Ludovico technique’). Rather than inducing nausea following Alex’s decision to commit a violent act, the revised Ludovico technique that undermined freedom of choice would serve to alter Alex’s emotional or motivational states in such a way that he would not decide to commit a violent act in the first place.

This difference is more than merely semantic, since it is not clear that interventions that restrict only an individual’s freedom of action would qualify as a ‘moral enhancement’ on some definitions of the term. For instance, such interventions would not qualify as moral enhancements on Douglas’ definition, according to which a moral enhancement is an intervention that can “reasonably be expected to result in (the recipient) having morally better future motives, taken in sum, than she would otherwise have had” (Douglas 2008). The recipient of an intervention that undermined their freedom of action might retain their immoral motives, and yet be unable to act in accordance with them.

A second ambiguity in Harris’ argument pertains to his understanding of the *value* of the freedom to fall, and more specifically, its relationship to responsibility, praise and virtue. Although it is clear that he takes this freedom to be morally significant, he often seems inconsistent with regards to his claims about *why* the freedom to fall matters. I have already explained that he understands autonomy to require the freedom to fall. He also claims that the possibility of virtue is contingent upon this freedom (Harris 2011, 104). These two claims, at least, seem compatible and perhaps even complementary. Furthermore, given the significant value commonly attributed to both autonomy and virtue, these claims imply that the freedom to fall is of moral significance. However, Harris’ understanding of the value of the freedom to fall becomes more elusive when we consider his remarks regarding the relationship of the freedom to fall to considerations of desert and moral responsibility.

There are two key claims that Harris makes in response to Persson and Savulescu’s criticisms of the freedom to fall argument that are worthy of our attention in this regard. First, in their critique of Harris, Persson and Savulescu (2012, 114) interpret him to be claiming that individuals who lack the freedom to fall cannot be morally praised for their actions. In response, Harris chastises Persson and Savulescu for presuming that the value of the freedom to fall is related to moral praiseworthiness in this way:

Savulescu’s and Persson’s presumptions here are well presumptuous. I am talking about freedom, not the state of the soul of the agent. What is morally important here is to be actually free … The issue is not praiseworthiness; it is liberty (Harris 2014, 248–249).

Second, Harris goes on to criticize Persson and Savulescu further, this time for their objection that the freedom to fall is not necessary for moral responsibility. To make this objection, Persson and Savulescu appeal to their famous God-Machine example. The God Machine is a Frankfurt-style form of moral enhancement, in which an omniscient machine intervenes when (and crucially, *only* when) individuals are about to perform a significantly immoral action, and changes their mind so that they do not perform it (Persson and Savulescu 2012). They claim that the God Machine would not affect the moral responsibility of agents who never chose to perform immoral actions (Persson and Savulescu 2012, 115). As such, they suggest that the God Machine example shows that Harris’ freedom to fall is not a necessary condition of moral responsibility. This, if true, is problematic for Harris’ argument because if what we want to maintain is moral responsibility, then it is not clear why we should worry about the freedom to fall.

However, Harris takes himself to be “… talking precisely about moral responsibility” in his arguments about the freedom to fall, and claims that the God Machine *would* (amongst other things) threaten the moral responsibility of agents who never chose to perform immoral actions, *contra* Persson and Savulescu (Harris 2014, 249). I shall flesh out Harris’ justification for this claim in the next section. At this point though, we can observe that his response to the God Machine example, taken together with some of the claims outlined above seemingly raise a puzzling tension in his view. The above analysis suggests that Harris believes that the freedom to fall is necessary for both autonomy and moral responsibility. Although he does not comment on the issue, moral responsibility is often plausibly understood to be closely related to considerations of desert; indeed, on some accounts moral responsibility is deemed to be necessary for desert.^3^ Yet, although Harris believes that autonomy and moral responsibility require the freedom to fall, he denies that he is talking about praiseworthiness, which is seemingly a desert-based attribute, or the state of the soul of the agent. This latter claim is even more puzzling when read in conjunction with his earlier claim that the freedom to fall plays an integral role in the possibility of virtue; after all, it seems that virtue must have something to do with ‘the state of the soul of the agent’, despite Harris’ claim that he is not talking about this.

In order to adequately assess Harris’ argument here, it is important to resolve these ambiguities, and to clarify the value of the freedom he defends, and its implications for moral responsibility, virtue and praiseworthiness. Of course, one way in which this tension could be resolved is if Harris denied the apparent connection between moral responsibility and desert (Feldman 1996). However, in the next section, I shall argue that it is possible to resolve the apparent tension in Harris’ view without making this controversial move. We can do so if we understand him to be invoking a broadly Aristotelian understanding of responsibility and praiseworthiness in these comments. I will suggest that this reading helps to explain why he thinks Persson and Savulescu’s famous God Machine objection fails, and how he might respond to another potential objection regarding the value of the freedom to fall.

## An Aristotelian Reading of the Freedom to Fall

In view of Harris’ invocations of the language of both freedom of action and freedom of choice, it should come as no surprise that his interlocutors have invoked different understandings of his objection in their critiques. For instance, Savulescu and Persson (2012, 406, emphasis added) claim that Harris “implies that moral enhancement would somehow make it impossible *to act* immorally”. In contrast, David DeGrazia writes “According to Harris, (moral bio-enhancement) would eliminate the freedom *to choose* to do wrong” (DeGrazia 2014, 365, emphasis added). Saskia Verkiel (2017) also seems to endorse this choice-focused interpretation of Harris, claiming that “Harris’ notion of freedom is characterised by “having options to do wrong”. In contrast, Christoph Bublitz (2016) astutely notices that we can understand Harris’ objection to be appealing to both freedom of choice and freedom of action. I agree with Bublitz’s assessment, but would add that a charitable reading of Harris would attribute this apparent lack of precision to an implicit assumption that freedom of action and freedom of choice (and their moral significance) cannot easily be disentangled. As I shall go on to explain, this is a key aspect of his understanding of the freedom to fall.

To illustrate that freedom of action and freedom of choice cannot easily be disentangled, we may observe that these freedoms, though distinct, are clearly related in important ways. First, even if an intervention that undermines the recipient’s freedom of choice leaves their freedom of action intact, the intervention may still be construed as rendering certain actions ‘undoable’ for the recipient in the way that Harris describes. The reason for this is that an agent will only be able to take advantage of the freedom of action to perform *x* if they are first able to form the motivating desire to perform *x* in the first place. Freedom of choice is, in this sense, prior to freedom of action. For example, if the revised Ludovico technique above made it impossible to choose to perform an immoral action, we might appropriately say that the recipient cannot perform immoral actions, even if, strictly speaking, his freedom of action has not been directly restricted.

Second, our beliefs about the extent of our freedom of action affect the way in which we come to make our choices. In *The Nicomachean Ethics*, Aristotle makes a similar point when he claims that rational choice is a deliberative desire for things *in our power* (Aristotle 2000, 1113a emphasis added). The thought here is that we consciously make our choices and decisions about what to do in the light of our beliefs about what we are able to do. Unless we are wholly sceptical about the possibility of freedom of choice, it seems that this sort of limitation is normally compatible with our choices being free; these beliefs can be understood as informing our choices, rather than undermining our freedom in making them.^4^

Accordingly, although freedom of choice and freedom of action can be understood to refer to different dimensions of the freedom to fall, it is perhaps not possible to entirely divorce them. More significantly for our purposes, it might also be argued that we cannot understand the moral significance and value of the freedom to fall if we treat freedom of choice and freedom of action in isolation.

On some views, however, it might be claimed that the value of freedom of choice *is* distinguishable from the value of freedom of action. Bublitz seems to implicitly endorse this kind of view, since he suggests that freedom to fall, understood as freedom of action, is not particularly valuable; its value can be outweighed by considerations of harm. For instance, Bublitz claims that we hypothetically ought to intervene to prevent a murder, even though this would involve restricting the prospective murder’s freedom to do evil (Bublitz 2016, 92). However, he also defends the claim that moral enhancement might threaten freedom of choice, and that this would be deeply problematic (Bublitz 2016, 92–94).^5^ In contrast, on Harris’ view, the value of the two freedoms is not as easily distinguished as this sort of analysis suggests, as the following passage makes clear:

Agents are quintessentially actors; to be an agent is to be capable of action. Without agency, in this sense, decision-making is … both morally and practically barren—literally without issue! (Harris 2014, 249)

Acknowledging that Harris interprets the value of freedom in this way suggests the following reading of the freedom to fall objection.^6^ First, it suggests how we might understand the apparently contradictory remarks that Harris makes with regards to moral praiseworthiness, virtue, and moral responsibility. Consider first the question of moral responsibility. The reason Persson and Savulescu’s ‘God Machine’ critique may not hit all the way home for Harris is that his accentuation of the importance of *action* (as well as decision-making) to agency suggests that he does not understand moral responsibility to pertain solely to making free choices. In order to be morally responsible in the way that concerns Harris, it is not sufficient to simply make free choices; those choices must also be able to result in action that actually makes a robust difference to external states of affairs. In fact, in a later commentary he insinuates that a form of freedom that does not allow for this (such as the freedom permitted by the God Machine) is hardly deserving of the name (Harris 2014, 253). *Pace* Savulescu and Persson then, the reason that the God Machine undermines moral responsibility for Harris is that the presence of the God Machine means that one’s decisions cannot have a robust effect on external states of affairs; good acts will be performed whether or not it is the agent herself who decides to perform them. The agent thus does not have what we might term ‘counterfactually sensitive responsibility for external states of affairs’. For brevity’s sake, I shall henceforth refer to this understanding of action and responsibility that Harris appeals to here as the ‘counterfactual external-state responsibility thesis’.

This understanding of moral responsibility has important implications for how we should interpret Harris’ understanding of the value of the freedom to fall and its relationship to considerations of praiseworthiness. For Harris, it seems that counterfactual external-state responsibility is central to our status as moral agents and achieving flourishing human lives. To see why, consider first Harris’ assertion that the possibility of virtue is contingent upon the freedom to fall, and his assertion that his arguments do not pertain to the ‘state of the soul of the agent’. Whilst seemingly contradictory, these two claims are reconcilable on an Aristotelian conception of virtue, a view that resonates with Harris’ action-focused understanding of moral responsibility and its value. For Aristotle, virtue of character, that is holding a disposition to act in accordance with virtue, is a result of habituation (Aristotle 2000, 1103a). Yet, virtue of character is not sufficient for attaining *eudaimonia*, the highest good for human beings; the reason for this is that eudaimonia is defined as a life of “*activity* of the soul in accordance with virtue” (Aristotle 2000, 1098a emphasis added). On this understanding, human flourishing, or the chief good for human beings, consists in the *use*, and not just the mere possession, of the disposition to act virtuously.^7^

This distinction has important implications for the relationship of virtue and eudaimonia to Aristotle’s conception of praiseworthiness, for whilst virtue of character is an appropriate subject of praise on the Aristotelian conception, (since it makes one the kind of person who will perform noble actions), eudaimonia itself is not an appropriate subject of praise. The reason for this is that praise can apply only to “things standing in relation to others”, and it thus cannot apply to the best of things (Aristotle 2000, 1101b). Accordingly, eudaimonia, a life of activity of the soul in accordance with virtue, is not to be praised, but rather *honoured*.

This Aristotelian understanding of the relationship between praise, virtue, and human flourishing can help to ease the apparent tension in Harris’ remarks regarding the value of the freedom to fall. Recall that for Harris, the God Machine is incompatible with what I have called counterfactual external-state responsibility. This is significant because, in addition to being a necessary condition for moral responsibility for Harris, such responsibility also seems to be necessary for the very possibility of living a life of *activity* of the soul in accordance with virtue. For Aristotle, this sort of life is necessary for human flourishing; for Harris, it seems that the possibility of living this sort of life is central to our very capacity for moral agency. 8

Therefore, with regards to the question of virtue and praiseworthiness, on this Aristotelian conception, Harris can be consistent when he claims that freedom to fall is necessary for virtue,^9^ that the God Machine undermines responsibility, but that he is not talking about praiseworthiness, or the state of the soul of the agent. It is not that the God Machine raises no issues about desert or about the state of the soul of the agent; rather, for Harris, there are bigger fish to fry than these issues in this context. The reason for this is that the God Machine threatens the possibility of agency, and with it the possibility of human flourishing. The issue is thus primarily one of Aristotelian honour rather than praise.

On this reading then, there is no contradiction in Harris’ remarks and his response to Persson and Savulescu’s critique. However, as I shall explain in the following section, even this consistent interpretation of Harris’ claims faces difficulties.

## The Parity Principle and the Value of Freedom

Resolving the ambiguities surrounding Harris’ understanding of the freedom to fall with this Aristotelian reading thus explains how he can offer a response to the God Machine objection, and also suggests a consistent interpretation of some of his apparently contradictory remarks. However, the freedom to fall objection still faces two other important criticisms.

First, Douglas has pointed out that we *already* employ non-biomedical moral enhancements that directly modulate emotional states, and these seem to be morally permissible. To illustrate, he provides two examples. The first involves stimulus avoidance:

Consider the case of a serial philanderer who, under the influence of unwanted sexual desires, repeatedly violates his relationship commitments, thus harming those near to him. Arguably, one way for such an individual to morally enhance himself would be to avoid situations that tend to elicit the unwanted sexual desires (Douglas 2013a, 162).

Douglas notes that such stimulus avoidance may prevent unwanted emotions from being elicited. The second example provides an illustration of a non-biomedical method of bringing about emotional change (rather than failing to elicit change):

Suppose I believe that I ought to be more moved by the plight of the global poor, and ought to do more to help them. However, I have trouble drumming up much sympathy for them. To remedy this, I set up my television so that it regularly displays disturbing and graphic images of the effects of poverty, though for such brief periods that I do not consciously recognize them. Nevertheless, through subliminal effects, the images increase my feelings of sympathy (Douglas 2013a, 163).

The thought here is that if we believe that these interventions are morally permissible then we should also regard NCMBEs as morally permissible.

In making this objection, Douglas seems to implicitly invoke the spirit of Neil Levy’s parity principle (Levy 2007a, b).^10^ The parity principle states that in assessing the moral permissibility of a neurointervention, we should ignore the nature of the intervention, and focus instead on the costs and benefits of the procedure, broadly construed. With respect to the debate surrounding NCBMEs, the parity principle implies that we ought to judge NCMBEs as morally on a par with non-biomedical interventions that might alter an agent’s moral judgments by directly modulating emotional states, unless we can establish that they differ with regards to their costs and benefits in a morally significant way.

Harris also implicitly accepts the dictates of the parity principle. However, he maintains that there *is* a morally relevant difference between NCMBEs and the interventions that Douglas mentions. The difference, he claims, is that the latter strategies involve a form of cognitive mediation. In support of his claim, Harris points out that in the case of stimulus avoidance, the intervention is a self-conscious strategy that combines understanding of cause and effect with modes of respect (including altruism and sensitivity) (Harris 2013, 170). In addition, the second example involves a strategy that has been deliberately chosen for reasons that are always available to the recipient (Harris 2013, 171).

However, this offers only a limited reply to the concern that Douglas raises. First, it is not clear why the *consensual* use of NCMBEs could not be described as a self-conscious strategy that combines understanding of cause and effect with modes of respect in the way that Harris stresses. Second, the cognitive mediation that these strategies purportedly involve according to Harris is merely derivative; it depends on the fact that agent *herself* has decided to employ these strategies. Yet, suppose that a third party imposed these strategies on another agent without consent; it is difficult to see how they would then incorporate cognitive mediation of the sort that Harris highlights. Furthermore, it is possible to provide examples of such externally imposed interventions that influence behaviour at a sub-conscious level (Douglas forthcoming). Consider the use of Baker-Miller pink in prison environments; following the publication of studies suggesting that mere exposure to this particular shade of pink can significantly reduce violent and aggressive behaviour, holding cells in a Naval Correctional Unit were for a period painted in this shade of pink, leading to a reduction in violent confrontations (Schauss 1979). The question we are left with is whether Harris would seek to incorporate this sort of environmental intervention into the set of cognitively mediated interventions that do not threaten freedom to fall, or into the set of interventions that would violate this freedom.

The inadequacy of Harris’ response to the challenge laid down by the parity principle is exacerbated when we take into account his broad understanding of moral responsibility. Even if Harris might deny that environmental interventions like Baker-Miller pink undermine freedom of choice, it is undeniable that we already employ an environmental intervention that takes away an individual’s freedom of action to perform immoral acts; namely, incarceration. This latter point is particularly telling since Harris (2014, 249) himself points out that “… prison is normally considered the antithesis of freedom”. Of course, whilst NCMBEs might only be imposed upon individuals in order to prevent them from re-offending, there are often other retributive and consequentialist reasons for why the state might be justified in incarcerating criminal offenders, in addition to the reason that we have to prevent them from carrying out further harm. Yet, not only is the latter a significant justification of incarceration, in some cases it is the *only* justification; those who endorse preventative detention schemes claim that we may incarcerate individuals who have already carried out a criminal sentence that satisfies retributive requirements on proportionate punishment, but who have been assessed as at high risk of re-offending (McSherry 2013; Gavaghan et al. 2014).

A natural reply for an opponent of NCMBEs to make here is to distinguish interventions that affect freedom of action from freedom of choice, and to argue that interventions that affect the latter are far more problematic. Whether or not this sort of response is plausible though, it is not one that Harris himself can make; after all, as the discussion in the previous section illustrates, freedom to fall incorporates freedom of action and freedom of choice in a highly interrelated manner. For Harris, the value of the freedom to fall cannot just be separated into the value of freedom of choice; the value of this freedom lies in the freedom of the choice, *in conjunction with* the freedom of action required for that decision to have an actual effect in the world. As such, in the absence of an explanation for why restricting freedom of action by restricting freedom of choice is worse than restricting freedom of action by other means, it seems that he must regard interventions that restrict freedom of action and interventions that restrict freedom of choice as equally problematic.

I shall return to Harris’ claims regarding the parity principle at the end of the paper. At this point though, let us turn our focus to another objection to the freedom to fall argument. The objection is that it is not clear that the freedom to fall is sufficiently valuable for Harris’ purposes. In most cases, we have very strong moral reasons to prevent individuals from being able to carry out acts of extreme violence, and it does not seem that taking away a person’s freedom to carry out such violence would be a great loss (DeGrazia 2014, 365; Savulescu and Persson 2012; Douglas 2013a; Hauskeller 2017; Bublitz 2016).^11^

Harris himself acknowledges this objection, and holds fast to the view that the value of liberty should take precedence in this ‘clash of values’, despite these concerns (Harris 2014, 257). Taken simply as a clash of values between liberty and public well-being, Harris’s position in this debate might be construed as fetishizing liberty. However, the above Aristotelian interpretation of Harris’ arguments perhaps suggests a more nuanced picture. On this analysis, the freedom of fall is not simply relevant to the well-being of individuals who might be construed as having a prudential interest in performing an immoral act;^12^ this freedom, insofar as it is necessary for virtue, is also necessary for the possibility of eudaimonia and moral agency for *anybody*, even those who do not take themselves to have any prudential reason to perform immoral actions.^13^

To this point, I have outlined the Aristotelian interpretation of Harris’ argument without criticism, in order to develop what I take to be the most nuanced, and consistent form of his freedom to fall objection. Although the Aristotelian reading offers a response to this third objection, I shall not pursue it further. My justification for turning to an alternative freedom-based objection is that even the Aristotelian interpretation remains problematic for a number reasons. First, it rests on the controversial counterfactual external-state responsibility thesis. This thesis can be criticised from at least two angles. Consider first that the thesis implicitly incorporates the view that moral responsibility requires that one has genuinely accessible alternative possibilities, or what Fischer and Ravizza have called ‘regulative control’ (Fischer and Ravizza 1998; Fischer 1999b). Even those who agree with Harris about the importance of regulative control for moral responsibility may nonetheless reject the counterfactual external-state responsibility thesis in the following way: They may argue that moral responsibility only requires that the agent has regulative control over whether it is *they themselves*, rather than a Frankfurtian intervention like the God Machine, that is the author of their action *x*; the agent need not, however, have regulative control over whether the act *x* would or would not be carried out *simpliciter* (which the presence of a Frankfurtian intervention *would* rule out).^14^ We may call the former responsibility counterfactual *internal-state* responsibility. Second, one may challenge Harris’ position more fundamentally, by denying that moral responsibility requires regulative control at all; it may only require what Fischer and Ravizza call ‘guidance control’, which requires only that the agent’s action flow from their own moderately reasons-responsive mechanism (Fischer and Ravizza 1998, 207; Fischer 1999b). Indeed, Frankfurt cases might be understood to provide motivation for this kind of view (Frankfurt 1969).

The Aristotelian interpretation of the freedom to fall also faces two further problems. The first is that the immoral actions of some can plausibly preclude the possibility of eudaimonia for others on the Aristotelian conception of virtue, since performing virtuous actions may require certain external resources (such as friends, wealth and beauty) of the sort that may be stripped from us by the immoral actions of others (Aristotle 2000, 1099b). Furthermore, on broadly Aristotelian views, the exercise of virtue is necessary, but not sufficient for eudaimonia;^15^ true flourishing might also require the presence of other good-making factors in one’s life, such as good health, or circumstances in which the end that one longs for is obtainable by virtuous means (Foot 2001, 95). If preventing others from committing seriously immoral acts can be construed as helping to preserve the external goods necessary for virtue, or other good-making factors in one’s life, it may in fact safeguard our ability to achieve eudaimonia.

Second, the conception of eudaimonia that is relevant to the freedom to fall objection on this Aristotelian interpretation is a highly moralized conception of well-being. As such, it is in tension with both hedonistic theories and those desire-fulfilment theories that do not incorporate objective elements. Accordingly, the appeal to the value of eudaimonia is unlikely to persuade either classical utiliarian or preference utilitarian advocates of moral enhancement who appeal to less objective accounts of well-being in grounding their moral theories. On such theories, it seems likely that the serious harms that moral enhancement might plausibly prevent would outweigh the prudential benefit of allowing individuals the freedom to fall.

To take stock, I have offered what I take to be the strongest and most consistent interpretation of Harris’ freedom to fall objection, and suggested that even this version of the objection faces important difficulties. However, this discussion has illuminated key problems for any freedom-based objection to NCMBEs. In light of the difficulties facing Harris’ argument, I shall, in the next section, consider an alternative freedom-based objection to moral enhancement that has not only developed as a response to the God Machine example, but which also aims to render plausible the idea that allowing immoral actions is a price worth paying for preserving the freedom that NCMBEs would violate.

## Moral Enhancement and Externalist Authenticity

In view of the problems with Harris’ freedom to fall approach, other critics have mounted an alternative freedom-based critique of moral enhancement. As I suggested above, one of the problems in analysing the freedom to fall argument is that it is presented somewhat ambiguously. In contrast, the argument that I shall outline in this section has developed in accordance with existing compatibilist approaches to moral responsibility in the philosophical literature. In order to better understand how this objection is situated, it will be useful to consider briefly some prominent landmarks in the philosophical landscape in this area.

One prominent compatibilist approach to understanding moral responsibility is to claim that for an agent to choose freely and to be responsible for their choice, they must choose on the basis of their *authentic* desires; on this approach, deciding how to act in accordance with one’s authentic desires is understood as a proxy for free choice and moral responsibility.

Naturally, this raises the question of what qualifies a desire as being authentic. To answer this question, it is useful to distinguish between internalist and externalist accounts of authenticity (Mele 1995, 146–147). According to internalist accounts, a desire is authentic if it satisfies some sort of internal psychological scrutiny. For example, Harry Frankfurt’s influential hierarchical theory of freedom may be understood as an internalist account of authenticity. For Frankfurt (1971), in order for an agent to freely decide to perform some action, they must endorse their first-order motivating desire to *x* with a second order volition that their first-order desire to *x* be effective in moving them to act. To illustrate, an unwilling drug addict who does not (at a second-order level) want her first-order desire to take drugs to be effective in moving her to act, lacks responsibility for her action if this desire moves her to act.

If we understand freedom of choice in this internalist sense, then it is not clear that NCMBEs would serve to undermine it, as they need not sever the relationship between one’s higher order volitions and motivating desires that internalist accounts emphasize; in fact, in some cases, they could serve to influence an agents motivation in such a way that they were better able to act in accordance with their authentic desires (Douglas et al. 2013).

In contrast, externalist accounts of authenticity potentially raise a problem for NCMBEs. Externalist accounts claim that the fact that a desire satisfies the agent’s internal psychological scrutiny is not sufficient for authenticity; on such accounts, authentic desires must also have a certain sort of causal history. The thrust behind externalist accounts is that it seems implausible to claim that agents who have been, say, psychologically manipulated or hypnotised, to form a motivating desire to x, have freely chosen to x, even if they now identify with that desire in accordance with conditions set out by internalist theories (Mele 1995). Problematically for proponents of NCMBEs, a plausible variant of externalism about authenticity claims that desires that are formed in a manner that bypasses the agent’s capacity for reason are inauthentic because of the nature of their causal history; and this seems to be just how NCMBEs would influence a recipient’s motivating desires (Bublitz 2016).

Although Bublitz (2016) is correct to note that ‘externalism’ is an influential version of compatibilism, it is not without its critics (Berofsky 1995). Moreover, not all versions of externalism raise problems for NCMBEs; indeed, in his defence of NCMBEs, David DeGrazia explicitly appeals to an externalist account of authenticity, according to which a person holds a motivating desire authentically if they identify with it, and this identification has not resulted primarily from influences that they themselves would, on careful reflection consider alienating (DeGrazia 2014). He suggests that in cases in which an NCMBE would influence an agent to act on the basis of first-order motivating desires with which they identify, the agent would welcome this influence, rather than find it alienating.

DeGrazia’s condition is a subjective externalist condition, insofar as his condition stipulates that is up to the agent *herself* [albeit an idealised counterfactual version of herself] to decide whether the aetiology of the desire is such that she should feel alienated from it.^16^ In view of the compatibility of subjective externalism with NCMBEs causing authentic desires, the opponent to NCMBEs is on stronger ground when they appeal to *objective* externalist conditions, according to which certain aetiologies undermine the authenticity of a desire, whether or not the agent herself believes that they do. For instance, on this approach it might be claimed that a desire is inauthentic if it is formed in a manner that bypasses the agent’s control over her own mental life (Mele 1995, 171).^17^ On this sort of account, ‘brainwashing’ undermines the individual’s responsibility, even if the target herself does not object to the aetiology of the desires she forms as a result of brain-washing.

Objective externalist conditions can be relational or non-relational. Non-relational conditions claim that certain causal histories (such as those that bypass the agent’s capacity for reason) undermine authenticity, whether or not that causal history incorporates third party control. On the other hand, relational objective externalism claims that only those causal histories that involve *third-party control* over the formation of one’s desires undermine authenticity. To illustrate the difference between non-relational and relational objective externalist theories, we may note that the former, but not the latter, allow for the possibility that non-agential forces (such as psychiatric disorders) can threaten the authenticity of one’s desires, if they cause agents to form desires in a way that bypasses their control over their own mental life.^18^

One appeal of such relational conditions is that they take the question of authenticity and freedom of choice out of the realm of the metaphysical, and into the more tangible realm of the political. Establishing whether an agent is choosing freely is not a matter of establishing that their will is free in some deep metaphysical sense; rather, it is a question of whether one agent has control over another’s desires.

In view of the above taxonomy of theories of authenticity, we may identify an alternative freedom-based objection to NCMBEs that has recently been proposed in the literature, as being grounded by an appeal to a relational, objective, externalist account of freedom. Robert Sparrow (2014) first outlined the form of the objection that I have in mind, and it is compatible with the sentiments of Bublitz’s (2016) remarks in his discussion of moral enhancement and the problem of manipulation; more recently however, this sort of objection has received its most sustained defence from Michael Hauskeller (2017).^19^

One of the attractions of this approach, and perhaps one of the main reasons this account has been invoked in the context of moral enhancement, is that it offers a way for opponents of NCMBEs to respond to the God Machine example, and Savulescu and Persson’s ensuing arguments. First, an individual who was prevented from performing an immoral act by the God Machine would clearly have had the freedom that externalists emphasise violated, even if the machine now causes them to internally identify with their ‘moral’ desire. This is a strike in favour of externalism over internalism.^20^ Just as importantly though, this form of externalism can also account for a further counter-example that Savulescu and Persson raise against the claim that moral enhancement undermines freedom or autonomy. As Savulescu and Persson rightly point out, “women are not less free than men because by biological nature they are more altruistic and less aggressive” (Savulescu and Persson 2012, 409); this, they claim is problematic for the freedom-based opponent of moral enhancement, since it seems such opponents must reject this claim if they hold that enhancing a man’s altruism (perhaps to the same level as women’s) would somehow render the recipient less free. Yet, this criticism cannot be levelled against the form of externalism under consideration here; such externalists can claim that women are *not* less free by virtue of their greater degree of altruism, and yet still maintain that interfering to increase men’s altruism would undermine the sense of freedom they have in mind.

Finally, A republican variant of the relational objective externalism objection can also claim, *contra* Savulescu and Persson, that even law-abiding citizens in the God Machine society would lack a valuable freedom (Sparrow 2013). The republican conception of freedom from non-domination stresses the importance of being free from dominating influences, whether or not those influences are actually exerted; to illustrate the importance of this freedom, consider a slave whose benign owner never prevents him from doing what he wants to do. For the republican, the fact that the slave-owner *could* prevent the slave from doing what he wants if he so chose is sufficient to establish that the slave is not free; the same could be said of members of a society in which the God machine operated (Pettit 1997).

The wide-spread discussion of an example that captures the imagination in the way that the God-machine does is hardly surprising. However, rather than serving to show that moral enhancement is (or is not) compatible with freedom or moral responsibility, the utility of the example is that it serves to illustrate the varied interpretations of the nature of freedom and moral responsibility that parties in this debate endorse. In this way, perhaps the example has served its purpose; it seems doubtful that these debates about the fundamental nature of freedom and moral responsibility will be resolved by continued discussion of whether the example itself is convincing. The divergence on this point merely reflects a more fundamental philosophical disagreement.

For this reason, I suggest that the focus of the debate surrounding moral enhancement and freedom should shift to the other two objections outlined in section II; the objection concerning the moral value of freedom, and the challenge laid down by the parity principle. Of all the proponents of the relational objective externalist freedom-based objection to moral enhancement, only Hauskeller has sought to provide an explanation as to why the value of this freedom is of such foundational importance that it can outweigh the value of preventing immoral actions. In the concluding section, I shall outline Hauskeller’s explanation, and consider its implications for the objection’s response to the challenge laid down by the parity principle.

## Moral Engineering and the Dilemma Posed by the Parity Principle

According to Hauskeller’s version of the objection under consideration, the reason that NCMBEs are problematic is that they involve third parties engineering an agent to think and feel a certain way, such that others have gained control over them. In order to explain why the violation of this sort of relational freedom is morally significant, Hauskeller appeals to the Habermasian claim that such “instrumentalization of nature might change the “ethical self-understanding of the species in such a way that we may no longer see ourselves as ethically free and morally equal beings guided by norms and reasons” (Hauskeller 2017). NCMBEs are thus likely to change the way in which we view ourselves and each other; more specifically, we might come to view human beings as unfree, as under the domination of another’s will. Yet, in becoming subjugated to this will, Hauskeller claims that human beings would thereby become ‘means to the end of morality’ rather than ends in themselves’ (Hauskeller 2017). NCMBEs would thus undermine a freedom that undergirds the whole enterprise of morality. In this sense, Hauskeller’s objection has a significant advantage over the freedom to fall objection; it can offer a coherent explanation as to why the freedom in question matters more than the prevention of significant harm to others.

Notably though, Hauskeller does not address the challenge laid down by the parity principle. In the absence of an explicit discussion of this challenge in Hauskeller’s remarks, it is prudent to turn to the Habermasian framework that the objection invokes. The framework is adopted from Habermas’ arguments against prenatal genetic enhancements. Liberals in the human enhancement debate, including Harris, have been heavily critical of Habermas’ arguments in this context (Buchanan 2011; Harris 2007; Agar 2004). Most strikingly for my purposes, one strategy that liberals adopt in replying to Habermas is to invoke a moral principle that is structurally similar to the parity principle in the neuroethics literature. The core claim of the parity principle is that the nature of an intervention *in itself* is not relevant to our judgements about its moral permissibility. In the context of prenatal genetic enhancements, it might similarly be claimed that genetic interventions and environmental interventions aimed at improving a child’s capacities should be treated alike, unless we can identify an ethically relevant difference between them with regards to their costs and benefits (Agar 2004). In considering Habermas’ arguments on this point, we can gain insight into how the argument from relational freedom might correspondingly respond to the challenge posed by the parity principle in the context of NCMBEs.

Habermas’ main argument for the claim that there is a moral difference between genetic and environmental enhancements is that only the latter involve *communicative action*. The concept of communicative action may broadly be understood to refer to action undertaken by agents involved in mutual deliberation aimed at a shared understanding, in the course of which each party reciprocally raises validity claims that can be accepted or contested (Habermas 1984, 99). For Habermas, our engagement in this sort of activity is central to our human dignity, to our understanding of each other as free and equal beings worthy of moral respect (Habermas 2003, 54). Human dignity is thus not a property that humans possess ‘by nature’, but rather indicates an inviolability that “… comes to have significance only in interpersonal relations of mutual respect” (Habermas 2003, 33). This understanding of the relationship between communicative action and human dignity has important implications for the moral permissibility of genetic enhancements. Whilst Habermas suggests that a neonate is treated as ‘one of us’ in the world of communicative actors (Habermas 2003, 35), prenatal genetic enhancements do not allow for the possibility of communicative action. Accordingly, genetic enhancements establish an asymmetry between the ‘designed’ individual and their ‘programmer’ (Habermas 2003, 64–65).

This putative difference between environmental and genetic enhancements is highly relevant to the context of moral enhancement. It seems plausible to claim that many seemingly permissible environmental forms of moral enhancement involve communicative action. Cognitive Behavioural Therapy (CBT) seems a paradigm example; in CBT the therapist and the recipient are engaged in a reciprocal dialogue that aims to dynamically evince a mutual understanding of the recipient’s thought patterns, how they contribute to their behaviour, and how these patterns might be changed; this in turn may lead to moral improvement. In contrast, it is not clear that NCMBEs would engage the offender in this form of communicative action; the change would be imposed upon the recipient, rather than adopted over the course of a reciprocal dialogue.

This appeal to communicative action seems to broadly echo Harris’ reply to the challenge posed by the parity principle. Recall that in responding to this challenge, Harris appeals to the moral significance of the fact that environmental enhancements involve cognitive mediation. *Pace* Harris, I have followed Douglas in suggesting that we already employ environmental interventions that non-cognitively improve moral behaviour in a way that seems to be morally permissible. Hauskeller’s appeal to communicative action goes beyond Harris insofar as it explicitly rules out the imposition of non-consensual environmental interventions (such as Baker-Miller pink) that amount to imposing a change upon the recipient, rather than persuading the individual to adopt a change over the course of a reciprocal dialogue. Yet, if this is so, should we thus conclude that the use of Baker-Miller pink violates relational freedom in the same way that an NCMBE would?

At this point, it becomes clear that one’s account of the value of the freedom putatively violated by NCMBEs will have significant implications for one’s response to the challenge laid down by the parity principle. If opponents of NCMBEs are to claim that these interventions are impermissible, then by the dictates of the parity principle they must either explain how seemingly permissible environmental interventions that directly modulate emotions differ from NCMBEs in a morally significant way, or they must accept that the permissibility of these two interventions stand or fall together.

As I explained above, one of the criticisms levelled at the freedom to fall objection is that it fails to adequately explain why the value of this freedom should take precedence over the value of preventing harm to others. I have suggested that the Aristotelian interpretation of Harris’ arguments may offer a response to this objection. Nonetheless, contrary to the Aristotelian interpretation, one benefit of admitting that the freedom to fall may *not* win out in a conflict with other values is that it makes the claim that NCMBEs violate this freedom more plausible, at least in light of the parity principle. The reason for this is that even if one claims that NCMBEs violate the freedom to fall, the seeming permissibility of environmental interventions that similarly seem to violate this freedom need not be problematic if the freedom in question is only of limited value. The violation of the freedom involved in either case might be deemed to be a price worth paying for the reduction in overall harm that it evinces.

In contrast, this sort of strategy becomes more and more problematic the higher the value we attach to the freedom that NCMBEs putatively violate. In the context of Hauskeller’s Habermasian argument (and the Aristotelian reading of Harris’ arguments about the value of freedom), if both NCMBEs and, say, Baker-Miller pink undermine freedom to the same extent, then proponents of these arguments are committed to the claim that *both* undermine either the ethical self-understanding of the species (on Hauskeller’s Habermasian framework), or the possibility of eudaimonia (on the Aristotelian understanding of the freedom to fall). Yet this seems problematic to say the least; in order to avoid this implication, one must either deny that NCMBEs threaten this freedom, or provide an alternative explanation for why NCMBEs violate this freedom in a way that externally imposed non-cognitive influences on our behaviour do not.

More generally, these reflections suggest that the parity principle and considerations pertaining to the moral value of freedom lead to a dilemma for freedom-based objections to NCMBEs. On the one hand, such an objection must show that NCMBEs violate a freedom that is sufficiently valuable to outweigh the moral value of preventing harms to others. On the other hand, the freedom in question must not be so valuable that seemingly permissible environmental interventions that have comparable effects on individuals’ behaviour amount to a violation of something so valuable that it should never be violated. My arguments above suggest that on the standard reading, the freedom to fall objection fails most obviously on the first prong, whilst the objection from relational freedom (and Harris’s argument on the Aristotelian reading I outlined in the first section of the paper) fails on the second.

Let me conclude by saying that I do not mean to deny that opponents of NCMBEs cannot escape this dilemma by offering an explanation of how NCMBEs differ in a morally significant sense from environmental influences. For example, one candidate morally relevant difference that has been proposed is that environmental influences are perceptually processed, and that “persons have most control over interventions whose sensual substrates they perceive” (Bublitz and Merkel 2014, 69). My purpose here has not been to assess this strategy. Rather, the point that I have tried to make is that many opponents of NCMBEs whose primary strategy is to establish that NCMBEs violate a morally significant freedom have largely misplaced their focus in seeking to respond to the God Machine example. In doing so, they are addressing a probably intractable question about the nature of moral responsibility itself, and, more importantly, they have failed to attend to the tangible challenge laid down by the parity principle, and its implications for one’s views concerning the value of freedom. Establishing that NCMBEs violate freedom may in fact be counter-productive to supporting one’s opposition to NCMBEs, if one does not supplement this argument with a plausible account of why seemingly plausible environmental interventions that directly modulate emotions or behaviour differ from NCMBEs in a morally significant way. In the absence of such an account, one’s arguments establishing the high moral significance of the freedom that NCMBEs would violate only serve to support the counter-intuitive conclusion that we have *already* violated, and indeed continue to violate, this freedom in our existing practices.

## Footnotes

1 See also (Fischer 1999a, 98). For a discussion of the relationship between the moral and legal senses of responsibility and how they pertain to the relationship between freedom and autonomy, see Garasic (2013).

2 See also Beehler (1982).

3 This sort of view can trace its lineage at least as far back as Aristotle’s discussion of praise and, voluntariness at the beginning of book III of the The Nicomachean Ethics. More recently, Saul Smilansky has defended the view that responsibility is a necessary condition of desert (Smilansky 1996). For criticism of Smilansky on this point, see (Feldman 1996) For a more recent analysis of the view that moral responsibility supports desert claims, see (King 2014).

4 Furthermore if an agent believes that they have extremely limited freedoms then this may have deleterious effects on their freedom of choice, by virtue of the phenomenon of adaptive preference formation (Colburn 2011). This might explain the plausibility of the claim that the Ludovico technique infringes Alex’s freedom of choice in some sense, (but in a less direct manner than other commentators have suggested).

5 Such a view is also implicit in accounts of autonomy that do not include conditions pertaining to freedom of action, in so far as these theories can be assumed to also hold that autonomy is valuable. See Christman (1991) and Ekstrom (1993).

6 I do not mean to suggest that the interpretation of Harris’ arguments I offer below is precisely representative of Harris’ own view. It is, however, one way of resolving his seemingly inconsistent claims. That said, his texts suggest that he endorses many of the central tenets of the reading I outline here, such as the counterfactual external-state responsibility thesis.

7 Virtue of character is thus a *hexis*, whilst eudaimonia is an *energeia*.

8 There is some agreement between my analysis and Verkiel (2017) here. However, on her moralized understanding, Verkiel suggests that the freedom that moral agency requires for Harris involves qualitatively different options. I endorse a broader interpretation; on my interpretation, the freedom that moral agency requires is outlined in the (non-moralized) counterfactual external-state responsibility thesis.

9 Notice that the issue may not be simply one of *moral* virtue. On the Aristotelian thesis of the reciprocity of the virtues, insofar as the absence of the freedom to fall rules out the possibility of moral virtue, it rules out the possibility of attaining *any* of the virtues.

10 I say only the spirit of the parity principle, since Douglas’ examples rely only on the intuition that the difference between subliminal images/stimulus avoidance and comparable biomedical enhancements are not morally significant. This does not commit him to the stronger claim embodied by the parity principle, according to which *only* costs and benefits can be morally relevant.

11 In his assessment of Harris’ argument (Bublitz 2016) goes further, arguing for the strong, broadly Kantian, clam that we do not have freedom of action to fall in a normative sense, since our normative freedoms are restricted by the freedom of others.

12 On both the Aristotelian and Socratic approach to virtue, one cannot have a prudential interest in acting immorally.

13 Verkiel (2017) seems to capture a similar claim when she writes that NCMBEs would only allow for amoral enhancement. Hauskeller criticizes Harris’ argument on the basis that the claim that virtue itself is important is only convincing on a theistic framework (Hauskeller 2017). Despite his appeal to Milton’s God, it is not clear that Harris understands himself to require this framework, given his secular approach to ethics. Moreover, the virtue ethicist can also claim that there is something to be to be gained by being good in the absence of God; the gain in question is in fact the highest good, namely eudaimonia.

14 For defences of this kind of view, see Mckenna (1997); Wyma (1997).

15 In contrast, the Platonic understanding of virtue claims that virtue is both necessary and sufficient for happiness.

16 See Christman (1991) for another subjective externalist account.

17 It should be noted that if the agent herself validly consents to an interventions that causes her to form a desire in a way that otherwise bypasses her mental control, this may still qualify as an authentic desire on the objective externalist approach; it will do so because the valid consent can be understood as the agent exerting mental control over her future mental state. See (Mele 1995, 171).

18 See Bublitz and Merkel (2009) particularly sections V-VIII for discussion of this difference, and a defence of a relational view.

19 Hauskeller himself does not use the language of freedom of choice or authenticity in delineating his objection, denying that there is such a thing as genuinely free choice (Hauskeller 2017). Instead he articulates the objection as being grounded by freedom from control. However, our difference in the use of these terms is only semantic; in denying free choice, Hauskeller is rejecting the ‘metaphysical’ accounts of free choice in favour of what I have termed relational, objective externalism regarding authenticity.

20 Note that Savulescu and Persson also acknowledge that an individual who was prevented from performing an immoral act by the God Machine would have had their freedom violated.
